# Normalized economical speed is influenced by aging and not by exercise habituation

**DOI:** 10.1186/s13104-023-06545-2

**Published:** 2023-10-05

**Authors:** Masahiro Horiuchi, Akira Saito, Kiyotaka Motoyama, Takehiro Tashiro, Daijiro Abe

**Affiliations:** 1 National Institute of Fitness and Sports in KANOYA, Shiromizu-1, Kanoya-shi, Kagoshima, 891-2393 Japan; 2 Center for Health and Sports Science, Kyushu Sangyo University, 2-3-1 Matsukadai, Higashi-ku, Fukuoka, 813-8503 Japan; 3 CNP Design, 4-1-5 Shimobaru, Higashi-ku, Fukuoka, 813-0002 Japan

**Keywords:** Locomotion, Gait, Step variability, Optimal speed, Froude number

## Abstract

**Objective:**

A U-shaped relationship between energy cost of walking (*C*_*w*_) and walking speed indicates that there is a specific speed minimizing the *C*_*w*_, called economical speed (ES). It is mostly slower in older adults than young adults; however, effects of leg length on the ES have been ignored. We investigated effects of aging and exercise habituation on the normalized ES by leg length (ES_normalized_). We quantified time delay of stride length and step frequency in sedentary young (SY), active young (AY), and active elderly (AE) adults in response to sinusoidal gait speed change at 30-s and 180-s periods with an amplitude of ± 0.56 m･s^− 1^.

**Results:**

The ES was significantly slower in the following sequence: AE, SY, and AY, whereas ES_normalized_ was slower in the AE than in other young groups, with no difference between AY and SY. AE and SY showed greater step variabilities at the 180-s period, whereas AY showed relatively smaller step variabilities at both periods. Collectively, the ES_normalized_ slowed due to aging, not due to exercise habituation. When optimizing the appropriate SL-SF combination for sinusoidal speed changes, young and elderly adults may adopt different strategies. Exercise habituation may reduce step variabilities in young adults.

## Introduction

The energy cost of walking per unit distance (*C*_*w*_; J·kg^− 1^·m^− 1^) presents as a U-shaped curve as a function of walking speed (*s*; m·s^− 1^) [1–3]. This indicates that every individual has a specific walking speed minimizing the *C*_*w*_ [1–13], referred to as economical speed (ES) [1–3]. A biological importance of the ES is that it is well associated with the preferred walking speed [13] in healthy populations [3–13].

Using some previous data, the measured ES (ES_measured_) apparently slowed in the elderly than in the young adults [1, 9–12]. Contrary findings were also presented in other studies [13–15]. Thus, it is controversial whether the individual ES slowed with aging. Different study settings (e.g., measured speeds), anthropometrics (height, obesity, or fitness level), or different calculation of the ES_measured_ render it difficult to obtain a consensus. Of these, anthropometrics, especially in leg length, should be reconsidered because longer leg length proportionally exhibited faster ES_measured_ [1]. Most of the previous studies have not normalized the ES_measured_ by leg length (ES_normalized_), although leg length in association with height was normally greater in the young adults than in the elderly adults [1, 9, 10, 12, 13]. Furthermore, daily exercise habituation can be a related factor. Previous studies have compared the ES_measured_ between active (or non-active) young and elderly counterparts [1, 13, 15]. Conversely, there is paucity in literatures comparing the ES_normalized_ between sedentary and active young adults. As the physical activity level is increased, the ES_measured_ is rendered faster in the elderly adults [14, 15]. Based on these previous findings, we hypothesized that the ES_normalized_ would be influenced by exercise habituation even in young adults. The primary objective of this study was to investigate the effects of aging and exercise habituation on the ES_normalized_ among active elderly (AE), active young (AY), and sedentary young (SY) adults.

When people walk at a given speed, continuous adjustment of their limbs is required to refrain from falling. This adaptation potentially contributes to minimize the *C*_*w*_ [16, 17], implying that the ability to adjust lower limb motions may be associated with aging and exercise habituation. Thus, sinusoidal speed changing protocol may be useful to manipulate the lower limb motions [18–21] as it requires the continuous adjustment of stride length (SL) and step frequency (SF). These continuous adjustments play a crucial role in safely performing our daily activities. Recently, we demonstrated that SL, but not SF, showed a likely delay in response to sinusoidal speed changes even in young females walking in high-heeled shoes [21]. Furthermore, peak ankle torque decreased with aging [22]. Consequently, we further hypothesized that gait-adjusting strategies may differ among the three groups. Therefore, the secondary objective of this study was to quantify the diversity of step variabilities among these three groups.

## Materials and methods

We used additional data from already published paper [19] based on an entirely different perspective. Seventeen SY, 16 AY, and 16 AE participated in this study (Table 1). Thirty-three healthy university students were classified between active and sedentary groups based on a recent guideline [23]. Elderly participants, aged over 65 years, were active members of the “Walking & Climbing Association of Fukuoka City.” All participants were nonsmokers, with no history of medication use or orthopedic and cardiorespiratory diseases. This study was approved by the ethical committee at Kyusyu Sangyo University (no. 2019-0002) and was performed under consideration of the Declaration of Helsinki. All participants signed written informed consent after being informed the purpose, experimental protocols, and possible risks.

**Table 1:** Physical characteristics in young and elderly participants

	Sedentary young (8M and 9F)	Active young (11M and 5W)	Active elderly (9M and 7F)	*F* values	*P* values
Age, years	20.3 ± 0.9	19.9 ± 0.6	74.1 ± 4.6^#^	2185	< 0.001
Height, m	1.613 ± 0.083	1.679 ± 0.068^*^	1.598 ± 0.087	4.713	0.014
Body mass, kg	57.2 ± 9.8	61.3 ± 9.3	58.6 ± 7.5	0.892	0.417
BMI, kg･m^− 2^	21.9 ± 2.9	21.6 ± 2.2	22.9 ± 2.0	1.196	0.312
Leg length, m	0.862 ± 0.059	0.901 ± 0.042^*^	0.852 ± 0.059	3.908	0.027
Exercise habituation	･None except physical education classes at their university	･Recreational sports (volleyball, soccer, tennis, track and field) ･90–120 min per day ･2–4 days per week	･Brisk walking (community walking and mountain club) ･40–60 min per day ･5–7 days per week		

Values are mean ± standard deviation. M, men; F, female; BMI, body mass index. ^*^ indicates significant vs. “Active elderly” and “Sedentary young”, and ^*#*^ indicates significant differences vs. “Sedentary and Active young” with Ryan’s *post-hoc* test

The participants visited our laboratory twice. On their first visit, they underwent a familiarization session on a treadmill (TKK3080, Takei Scientific Instruments Co. Ltd., Niigata, Japan) at several speeds without grasping handrails of the treadmill during walking. Subsequently, we determined their individual preferred walking speed [13]. Following a 10–15 min seated rest period, they performed the ES_measured_ determination protocol at 6–7 different gait speeds. These speeds were incrementally set at 0.44-0.67-0.89-1.11-1.33-1.56 m·s^− 1^ (elderly women), -1.67 m·s^− 1^ (elderly men), -1.78 m·s^− 1^ (young women), and − 2.00 m·s^− 1^ (young men) [1]. Each speed was maintained for 4-min. Oxygen uptake (VO_2_) and carbon dioxide (VCO_2_) were continuously measured using a breath-by-breath technique (AE310-S, Minato Medical Science, Osaka, Japan). To calculate the *C*_*w*_, an average VO_2_ and VCO_2_ for the final 2-min at each speed was used [24].

$${C_w}\left( {J \cdot k{g^{ - 1}} \cdot {m^{ - 1}}} \right) = \frac{{4.186 \times \left( {3.869 \times {\text{VO}_{2}} + 1.195 \times {\text{VCO}_{2}}} \right)}}{s}$$

A U-shaped relationship between *C*_*w*_ values and gait speeds was approximated with a quadratic Eqs. [1–3]:

$${C_w}\left( s \right){\text{ }} = {\text{ }}a \cdot {s^2} + b \cdot s + c$$

where the coefficients a, b, and c are determined by the least squares methods. The ES_measured_, at which the U-shaped *C*_*w*_-s relationship becomes minimal, can be obtained when the *C*_*w*_’ (*s*) is zero [1–3]. Thus, the ES_measured_ was calculated by a following equation:

$$E{S_{measured}} = \frac{{\left| { - b} \right|}}{{2a}}$$

The ES_normalized_ was calculated on the “dynamic similarity” theory [25], providing that geometrically similar legged locomotion will walk similarly at the same Froude number defined as:

$$Froude\,number = \frac{{({\text{E}}{{\text{S}}_{{\text{measured}}}})^2}}{{g \times {\text{leg length}}}}$$

where *g* is the gravitational acceleration (9.81 m·s^− 2^). Energetically optimal gait speed can be obtained when the Froude number is 0.25 [25]. Thus, the ES_normalized_ was calculated as follows:

$$E{S_{normalized}} = \frac{{0.3193 \times {\text{E}}{{\text{S}}_{{\text{measured}}}}}}{{\sqrt {{\text{leg length}}} }}$$

One week later, biomechanical measurements were conducted on the second visit. To capture motion data, eight high-speed cameras (Kestrel300, MAC3D System, Rohnert Park, CA, USA) were set with a sampling rate of 100 Hz [20]. The participants walked at their preferred walking speed in young adults or 90% preferred walking speed in elderly adults for 30-s, thereafter, the treadmill speed was sinusoidally controlled at 30-s and 180-s periods with an amplitude of ± 0.56 m·s^− 1^ (± 2 km·h^− 1^) in a randomized order with 5-min interval. The motion data were used to determine the time delay (TD) of the SL and SF against sinusoidal speed change. The SL and SF were approximated using the following equation:

$$SL{\text{ }}and/or{\text{ }}SF{\text{ }} = {\text{ }}A \cdot sin{\text{ }}(\omega t - TD)$$

where A, ω, and *t* represent amplitude, angular frequency, and time (msec), respectively.

Values are mean ± standard deviation. One-way analysis of variance (ANOVA) was used for comparisons in physical characteristics, ES_measured_, and ES_normalized_ among three groups, respectively. Two-way (3 groups × 2 sinusoidal periods) repeated measures ANOVA was used for comparisons of the TD of SL (TD_SL_) and SF (TD_SF_). When *F* values were significant, Ryan’s *post-hoc* test, which can be used regardless of data distribution [26], was used for further analyses. The statistical significance was set at *p* < 0.05.

## Results

Height and leg length in the AY group were greater than the other groups with no differences in body weight and body mass index among the groups (Table 1).

**Fig. 1:**
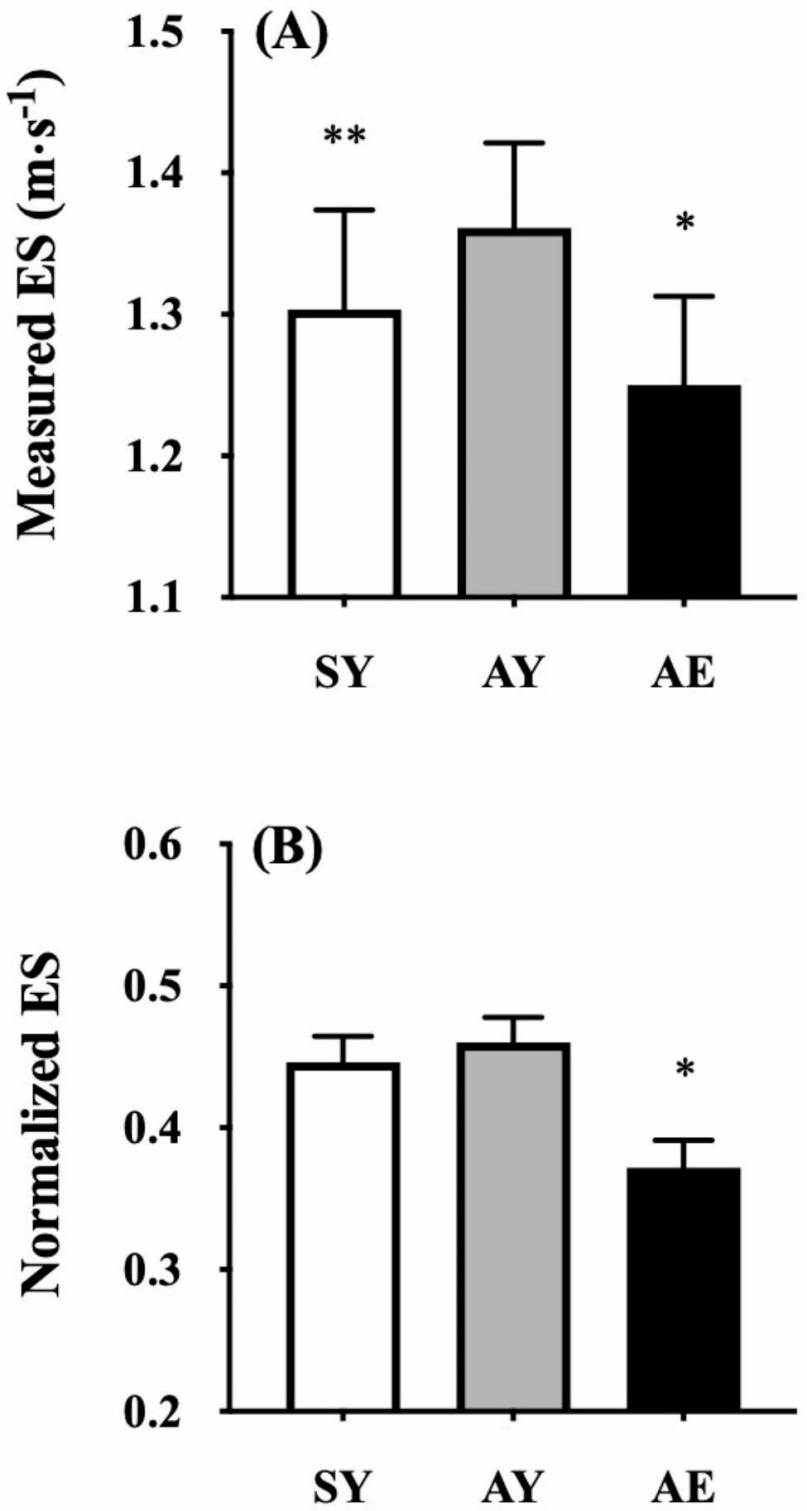
Comparisons of measured (A) and normalized (B) economical speed (ES) between sedentary young (SY; white bars), active young (AY; gray bars), and active elderly (AE; black bars) adults, respectively. Values are mean ± standard deviation. ^*^p < 0.05 between AE and SY and between AE and AY for both ES_measured_ and ES_normalized_. ^**^p < 0.05 between SY and AY for ES_measured_.

The ES_measured_ was fastest in the AY group (1.361 ± 0.058 m·s^− 1^), followed by the SY group (1.304 ± 0.068 m·s^− 1^) and AE group (1.250 ± 0.061 m·s^− 1^), with significant differences among the groups (*F* = 11.781, *p* < 0.001, Fig. 1A). The ES_normalized_ was significantly slower in the AE group (0.433 ± 0.021) compared to that in the SY group (0.448 ± 0.018; *t* = 2.303, *p* = 0.026) and AY group (0.458 ± 0.018; *t* = 3.658, *p* < 0.001), with no significant difference between SY and AY (*t* = 1.410, *p* = 0.165) (Fig. 1B).

**Fig. 2:**
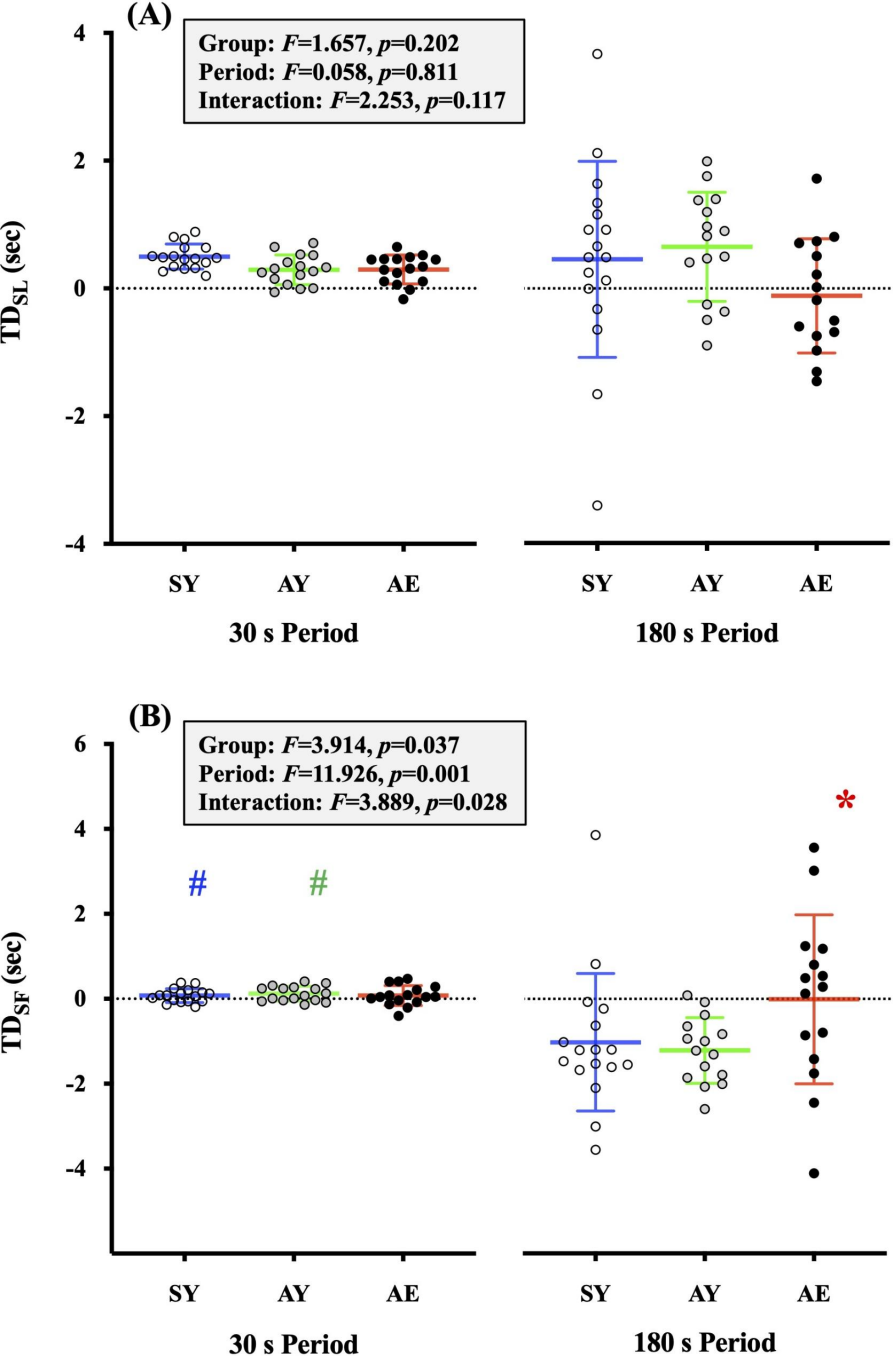
Time delay of stride length (TD_SL_) and step frequency (TD_SF_) at 30-s and 180-s period among SY, AY, and AE groups. ^*^significantly greater TD_SF_ in the AE than in the AY and SY at the 180-s period. ^#^significant difference in the TD_SF_ between 30-s and 180-s within the same groups (within SY or AY, respectively). Two participants (AE = 1 and AY = 1) were excluded due to a data unavailability at the 180-s period

There were no significant main effects of group (*F* = 1.657, *p* = 0.202), period (*F* = 0.058, *p* = 0.811), or interaction effects (*F* = 2.253, *p* = 0.117) in the TD_SL_ (Fig. 2A). There was a significant interaction effect in the TD_SF_ (*F* = 3.889, *p* = 0.028, Fig. 2B), along with main effects of group (*F* = 3.914, *p* = 0.037) and period (*F* = 11.926, *p* = 0.001) (Fig. 2B). A simple main effect of period showed that the TD_SF_ in the SY and AY groups at the 30-s period were significantly lower than those at the 180-s period, respectively (*F* = 7.821, *p* = 0.001 in the SY and *F* = 11.819, *p* = 0.001 in the AY). A *post-hoc* test further revealed that the TD_SF_ in the AE group at the 180-s period was significantly lesser than in the SY group (*t* = 3.094, *p* = 0.004) and AY (*t* = 3.497, *p* < 0.001) (Fig. 2B).

## Discussion

On average, a relatively smaller difference was observed in the ES_normalized_ (~ 3.9%; Fig. 1B) than the ES_measured_ (~ 6.2%; Fig. 1A) between AE and the other two groups, suggesting that leg length plays a key role in determining the individual ES. Additionally, no significant difference was observed between SY and AY in the ES_normalized_ (Fig. 1B), indicating that exercise habituation did not affect the ES_normalized_, at least, in young adults. Thus, the first hypothesis was rejected. An age-related upward shift of the *C*_*w*_-*s* curve is normally accompanied with a leftward shift of that curve, resulting in a slower ES_measured_ [1, 9, 12]. It has been considered by a longer thigh muscle co-activation period in a gait cycle [9, 11, 27–29], which would be a trade-off between gait stability and energy expenditure during walking in the elderly adults. It was noteworthy noting that the *C*_*w*_-*s* curve and/or *C*_*w*_ at some selected gait speeds was lower in elderly distance runners than in elderly habitual walkers [13–15, 30]. Remarkably, compared to healthy young adults, neither leftward nor upward shifts of the U-shaped curve were observed in elderly runners [14] and cyclists [15] compared to healthy young adults. These aerobic exercises can mitigate age-related upward and leftward shifts in the *C*_*w*_-*s* curve [14, 15], thereby potentially avoiding a decline in the ES. Some considerations are still necessary because cycling is not a bipedal locomotion. Running exercise requires much faster optimization of the SL-SF combinations compared to walking. Notably, cycling also requires quick steering to maintain two-wheeled posture. Thus, such a postural adjustment ability during cycling may extend to gait stability because quick optimization of the SL-SF combinations can reduce *C*_*w*_ [16, 17]. Both previous studies and our current findings suggest that habitual exercise may be able to mitigate age-related deterioration of the individual ES.

Step width of the AE in our original study was not different from that of younger counterparts [20]. Instead, elderly adults normally present a shorter SL compared to the young adults [31]. In that case, a faster SF is necessary because gait speed should correspond to the product of the SL and SF. During sinusoidal speed changing condition, quick optimization of the SL and SF is continuously required to catch up with the speed change. In support of our second hypothesis, we observed that the variability of TD_SL_ in the SY group (Fig. 2A) and TD_SF_ in the AE group (Fig. 2B) was notably high at the 180-s period. Variability in physiological responses during exercise, such as heart rate variability, typically reflects exercise tolerance [32]. Therefore, these greater variabilities may indicate a greater locomotive flexibility to optimize SL-SF combinations in response to passive gait speed changes. However, excessive gait variability is associated with an increase in fall risks [33] and *C*_*w*_ [16, 17]. Thus, these greater variabilities of TD_SL_ in the SY group (Fig. 2A) and TD_SF_ in the AE group (Fig. 2B) at the 180-s period suggest that strategies for optimizing appropriate SL-SF combinations against sinusoidal speed changes differ between SY and AE. That is, SY adopted by manipulating SF (in particular, by preceding the SF), whereas AE adopted by manipulating SL. In contrast, AY exhibited relatively smaller variabilities in TD_SL_ and TD_SF_ at both periods (Fig. 2A and B), indicating that exercise habituation tends to reduce step variabilities in young adults.

## Limitations

Technological limitations should be stated. Two comparative studies presented phase shift in degree [18, 19], equivalent to the TD_SL_ and TD_SF_ in the present study. Surprisingly, both TD_SF_ and TD_SL_ in healthy young adults showed negative values over 4-s in SF and 1.5-s in SL even at 60-s sinusoidal speed changing protocol [18, 19]. However, the trend of our results completely different from those of our previous studies (Fig. 2). This could be attributed to the different calculation techniques. Both previous studies used interpolated 1-s data for calculating the phase shift of the SF and SL. That is, TD_SL_ and TD_SF_ values with a sampling frequency of 1.0 Hz were treated based on the discrete Fourier transform. If SF and/or SL variabilities occurred above 0.5 Hz that corresponded to the Nyquist folding frequency of the original sampling frequency (1.0 Hz), those variabilities could contaminate low-frequency spectrums, so-called “aliasing” [34].

## Data Availability

The datasets used and/or analyzed during the current study are available from the corresponding author on reasonable request.

## Abbreviations

AE: active elderly
ANOVA: analysis of variance
AY: active young
*C*_*w*_: energy cost of walking
ES_measured_: measured economical speed
ES_normalized_: normalized economical speed
*s*: speed
SD: standard deviation
SF: step frequency
SL: stride length
SY: sedentary young
TD: time delay
TD_SF_: time delay of step frequency
TD_SL_: time delay of stride length
VO_2_: oxygen uptake
VCO_2_: carbon dioxide output
